# Triamcinolone acetonide-loaded nanoparticles encapsulated by CD90^+^ MCSs-derived microvesicles drive anti-inflammatory properties and promote cartilage regeneration after osteoarthritis

**DOI:** 10.1186/s12951-022-01367-z

**Published:** 2022-03-19

**Authors:** Yuanlong Li, Qingqiang Tu, Dongmei Xie, Shurui Chen, Kai Gao, Xiaochun Xu, Ziji Zhang, Xifan Mei

**Affiliations:** 1 Department of Orthopedics, The First Affiliated Hospital of Jinzhou Medical University, Jinzhou, China; 2 Department of Orthopedics, The First Affiliated Hospital, Sun Yat-Sen University, Guangzhou, China; 3 The Third Affiliated Hospital, Sun Yat-Sen University, Guangzhou, China

**Keywords:** Osteoarthritis, Biomimetic nanoparticles, CD90^+^ MCSs-derived micro-vesicle, Cartilage regeneration, Anti-inflammatory

## Abstract

**Background:**

Osteoarthritis (OA) is a highly prevalent human degenerative joint disorder that has long plagued patients. Glucocorticoid injection into the intra-articular (IA) cavity provides potential short-term analgesia and anti-inflammatory effects, but long-term IA injections cause loss of cartilage. Synovial mesenchymal stem cells (MSCs) reportedly promote cartilage proliferation and increase cartilage content.

**Methods:**

CD90^+^ MCS-derived micro-vesicle (CD90@MV)-coated nanoparticle (CD90@NP) was developed. CD90^+^ MCSs were extracted from human synovial tissue. Cytochalasin B (CB) relaxed the interaction between the cytoskeleton and the cell membranes of the CD90^+^ MCSs, stimulating CD90@MV secretion. Poly (lactic-co-glycolic acid) (PLGA) nanoparticle was coated with CD90@MV, and a model glucocorticoid, triamcinolone acetonide (TA), was encapsulated in the CD90@NP (T-CD90@NP). The chondroprotective effect of T-CD90@NP was validated in rabbit and rat OA models.

**Results:**

The CD90@MV membrane proteins were similar to that of CD90^+^ MCSs, indicating that CD90@MV bio-activity was similar to the cartilage proliferation-inducing CD90^+^ MCSs. CD90@NP binding to injured primary cartilage cells was significantly stronger than to erythrocyte membrane-coated nanoparticles (RNP). In the rabbit OA model, the long-term IA treatment with T-CD90@NP showed significantly enhanced repair of damaged cartilage compared to TA and CD90^+^ MCS treatments. In the rat OA model, the short-term IA treatment with T-CD90@NP showed effective anti-inflammatory ability similar to that of TA treatment. Moreover, the long-term IA treatment with T-CD90@NP induced cartilage to restart the cell cycle and reduced cartilage apoptosis. T-CD90@NP promoted the regeneration of chondrocytes, reduced apoptosis via the FOXO pathway, and influenced type 2 macrophage polarization to regulate inflammation through IL-10.

**Conclusion:**

This study confirmed that T-CD90@NP promoted chondrocyte proliferation and anti-inflammation, improving the effects of a clinical glucocorticoid treatment plan.

**Graphical Abstract:**

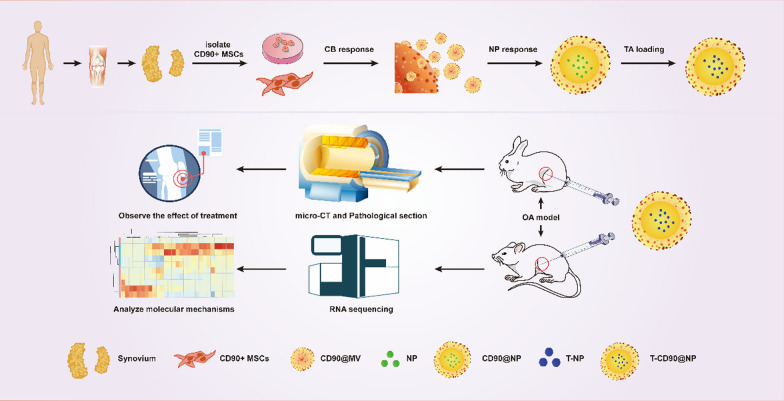

**Supplementary Information:**

The online version contains supplementary material available at 10.1186/s12951-022-01367-z.

## Highlights

- Long-term injection of glucocorticoids in the knee joint cavity promotes loss of cartilage content.
- CD90-positive stem cell vesicles encapsulated with Triamcinolone acetonide-loaded nanoparticles have good materials.
- T-CD90@NPs drive anti-inflammatory properties and promote cartilage regeneration after osteoarthritis.
- T-CD90@NPs regulate the polarization of type 2 macrophages to resist inflammation.
- T-CD90@NPs promote chondrocyte regeneration through the FOXO signaling pathway.

**Supplementary Information:**

The online version contains supplementary material available at 10.1186/s12951-022-01367-z.

## Introduction

Osteoarthritis (OA) is a highly prevalent human degenerative joint disorder that causes pain and dysfunction, seriously endangering physical and mental health. Worldwide, OA affects 250 million people [1]. The disease causes cartilage injury, osteophyte formation, vascular invasion of the articular surface, and synovial inflammation. The confirmed OA risk factors include obesity, aging, sex, previous joint injury, genetics, or anatomical factors, but the exact pathogenesis of OA is unclear [2, 3]. The pathological mechanisms of OA include an increase in matrix-degrading enzymes, for example, MMP13, extracellular matrix (ECM) degradation, reductions in SOX9-related transcription factors, and chondrocyte apoptosis [4]. Therefore, the current OA clinical treatment mainly involves injecting drugs into the intra-articular (IA) cavity and surgery. Unfortunately, these treatment strategies are ineffective. Thus, optimized, effective treatments are urgently required.

Early OA clinical treatments involve injecting glucocorticoids and sodium hyaluronate into the joint cavity [5, 6]. Short-term glucocorticoid use is beneficial for anti-inflammatory effects and pain relief. However, long-term glucocorticoid use may cause chondrocyte cycle arrest and cartilage loss [7–11]. Therefore, the anti-inflammatory and analgesic ability of glucocorticoids in OA treatment remains limited unless the problem of chondrocyte cycle arrest can be solved. Another OA treatment to address glucocorticoid limitations employs mesenchymal stem cells (MSCs), which induce proliferation, differentiate into cartilage, and transport drugs [12, 13]. MSCs promote cartilage proliferation and restart the cartilage cell cycle in OA treatment, but the anti-inflammatory and analgesic ability of MSCs is weak [14].

Nanoparticle-based biomimetic drug delivery systems (DDSs) are another OA treatment strategy with fewer side effects [15]. The combination of the natural cell membrane and synthetic NPs disguise NPs as endogenous cells, reducing their elimination and prolonging therapeutic effects [16]. In this system, NPs are loaded with drugs, and the encapsulated biological cell membrane exerts cellular biological effects [17, 18]. The most important factor in preparing biological cell membranes is to wrap the NPs [19]. This NP-based DDS allows glucocorticoids to retain their anti-inflammatory and analgesic effects without affecting the proliferation of cartilage cells.

We used synovial-derived MSCs to encapsulate glucocorticoid-carrying NPs. However, not all synovium-derived-MSCs promote cartilage proliferation [20, 21]. Reports demonstrated that the number of human synovial CD90-positive MSCs (CD90^+^ MSCs) was significantly reduced in OA and that CD90^+^ MSCs may be involved in cartilage repair in OA [22–24]. Similarly, micro-vesicles extracted from CD90^+^ MSCs (CD90^+^@MV) promoted cartilage repair [25]. Unlike the traditional membrane extraction method, CD90^+^@MV benefits from density gradient centrifugation and ultracentrifugation [26, 27]. The CD90^+^@MV cell membrane extracted in this process is rich in CD90 proteins. Membrane proteins play important roles in bio-functions.

**Scheme 1:**
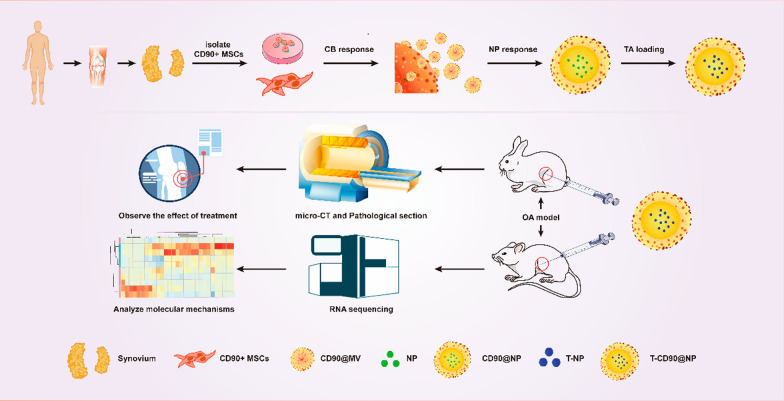
Schematic illustration of the entire study

In this study, CD90^+^@MV was prepared to wrap NPs loaded with TA, a common clinical glucocorticoid for knee joint injection in OA. The CD90^+^@MV-coated NPs containing TA (T-CD90^+^@NPs) were developed with higher TA concentrations than could be achieved by the capacity of bare NPs. CD90^+^@NP uptake by cartilage cells was better than that of red blood cell membrane-coated NPs (RNP). This study determined the therapeutic efficacy of T-CD90^+^@NP in rabbit OA treatment using micro-CT and pathological staining analysis. The anti-inflammatory ability of T-CD90^+^@NP was tested in the early OA stage, and the ability to promote the proliferation of chondrocytes during the late OA stage in the rat model. Finally, the anti-inflammatory and proliferation mechanisms were determined using transcriptomic (RNA-seq)-analysis of a rat model. This study synthesized T-CD90^+^@NP for the first time and proved the ability of T-CD90^+^@NP to induce TA-promoted chondrocyte cycle arrest while retaining the anti-inflammatory effect of TA. This approach may optimize traditional glucocorticoid injection for treating joint cavities in OA (Scheme 1).

## Results

### TA has limited long-term efficacy and causes chondrocyte cell cycle arrest in human OA

**Fig. 1:**
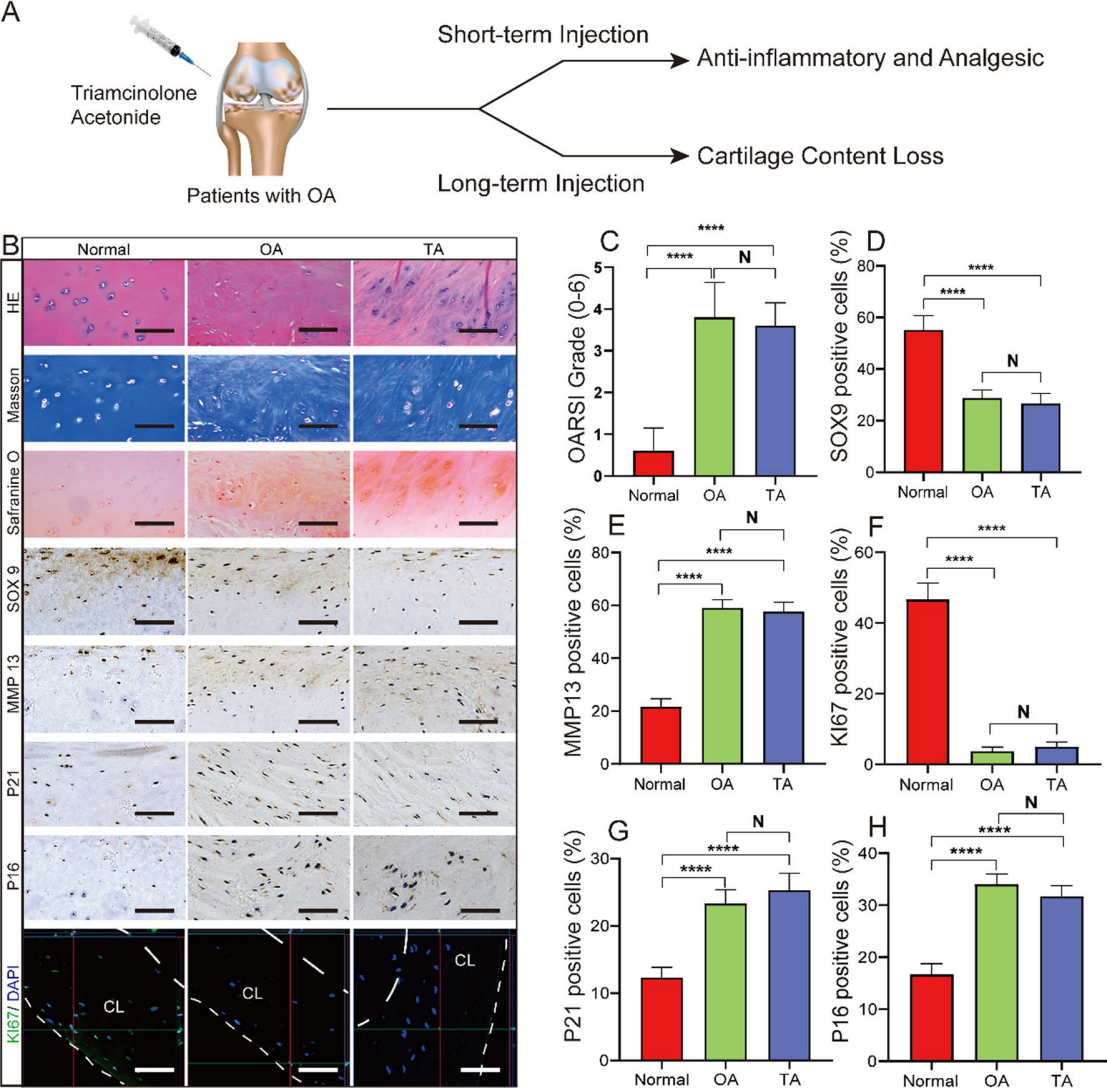
Pathological detection of TA IA in patients with OA. **A** Pattern diagram. **B** H&E, Safranin O, and Masson stain. Immunohistochemical staining of SOX9, MMP13, P16, P21, and Ki67 of knee cartilage in the different groups. The cartilage layer (CL) is between the white dotted lines. Bar = 100 μm, n = 5 (**C**–**H**). Statistical analysis of OARSI scores, SOX9, MMP13, Ki67, P21, and P16. ****P < 0.0001, N = no statistical difference

The knee cartilage of OA patients who had received 3–4 IA TA treatments for over one year before testing was used to observe the long-term efficacy of TA in human OA patients. Histomorphological staining objectively proved the condition of tissue repair because the articular cartilage layer is defective in OA. As the disease progresses, the defect is filled with new cartilage or fibrous connective tissue. Hematoxylin and eosin (H&E) and Masson staining (Fig. 1B) showed that the surface fibrillation area was larger, and the chondrocytes were abnormally distributed in the OA and TA groups compared to the normal group.

Safranin O fast green staining showed that the cartilage polysaccharide levels were reduced, and the cartilage matrix was severely degraded in the OA and TA groups compared to the normal group. However, there was no substantial difference in cartilage damage between the OA and TA groups. Quantitative analysis using OARSI scoring revealed high scores, which indicated poor tissue repair. The results showed that the OA and TA groups had significantly higher scores than the normal group, but the scores between the OA and TA groups were not substantially different (Fig. 1C).

The SOX9 and MMP13 immunohistochemical (IH) staining determined the proliferation, differentiation, and degradation of chondrocytes. The results showed that SOX9 expression in the cartilage layer was significantly decreased, but MMP13 was increased in the OA and TA groups compared to the normal group. However, both SOX9 and MMP13 expressions were not substantially different between the OA and TA groups (Fig. 1D, E). Cell cycle-related factors P21, P16 (negative cell cycle regulator), and Ki67 (cell proliferation and regeneration marker) were detected to observe chondrocyte cell cycle arrest after TA treatment. The results showed that P21 and P16 expression significantly increased in the cartilage layer, but Ki67 considerably decreased in the OA and TA groups compared to the normal group. There was no significant difference in the expression of P21, P16, and Ki67 between the OA and TA groups (Fig. 1F–H). Altogether, these results revealed that in the long-term, TA treatment caused cartilage tissue structure disorder and chondrocyte cell cycle arrest in OA patients.

### Extraction and activity of CD90-positive stem cells from human synovium

**Fig. 2:**
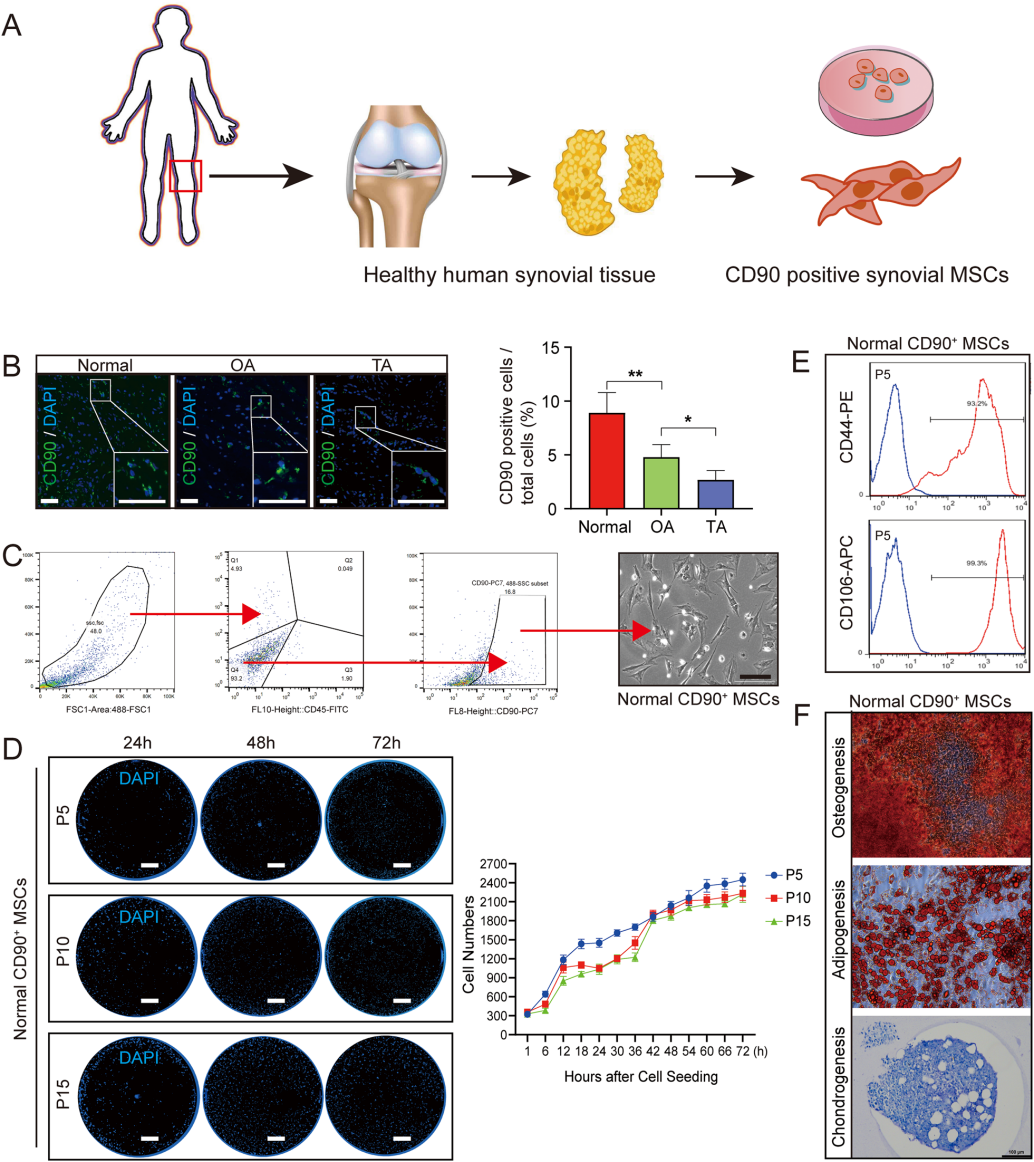
Extraction and identification of CD90^+^ MSCs from human synovium. **A** Pattern diagram. **B** CD90 (green) and DAPI (blue) immunofluorescence staining of human synovium in the different groups, Bar = 100 μm, n = 5. **C** CD90^+^ MSCs isolated from normal synovium by flow cytometry, with CD45 as an exclusive marker. Cultured cells were tested for spindle morphology and adherence on the plate. **D** Representative DAPI-stained images and proliferation curves of CD90^+^ MSCs (passages 5/10/15) based on cell numbers from 0 to 72 h. **E** The expression of cell surface markers (CD44 and CD106) on CD90^+^ MSCs detected by flow cytometry. **F** The representative stained images show that CD90^+^ MSCs differentiated into osteocytes (Alizarin red), adipocytes (oil red O), and chondrocytes (toluidine blue). *P < 0.05, **P < 0.01

The human synovium immunofluorescence (IF) staining results showed that CD90 expression was significantly higher in the normal group than in the OA group, whereas the expression was decreased in the TA group (Fig. 2B). This observation indicated that TA treatment continually reduced the number of CD90-positive cells in OA synovium. Thus, we could infer that CD90^+^ MSCs from the synovium were related to TA-induced chondrocyte cell cycle arrest in OA. We extracted CD45 (FITC) negative and CD90 (PC7) positive cells from healthy human synovium via fluorescence-activated cell sorting based on adherent growth on the plate and spindle morphology (Fig. 2C). Next, the proliferative ability of the obtained cells (normal CD90^+^ MSCs) was tested through different generations (P5, P10, and P15). The DAPI staining results showed that the cells increased from ~ 300 to ~ 2500 within 72 h, but the cell proliferation ability was not different between the generations (Fig. 2D). In addition, normal CD90^+^ MSCs expressed CD44 (93.2%) and CD106 (99.3%), representing common MSCs surface markers (Fig. 2E). Inducing normal CD90^+^ MSCs in osteogenic, adipogenic, and chondrogenic conditioned media verified their differentiation ability. The results showed that Alizarin Red, oil red O, or toluidine blue positively stained the differentiated normal CD90^+^ MSCs (Fig. 2F). Altogether, these results suggested that CD90^+^ MSCs from healthy human synovium had good proliferation and differentiation ability.

### Characterization of CD90@NP and its uptake ability by cartilage

**Fig. 3:**
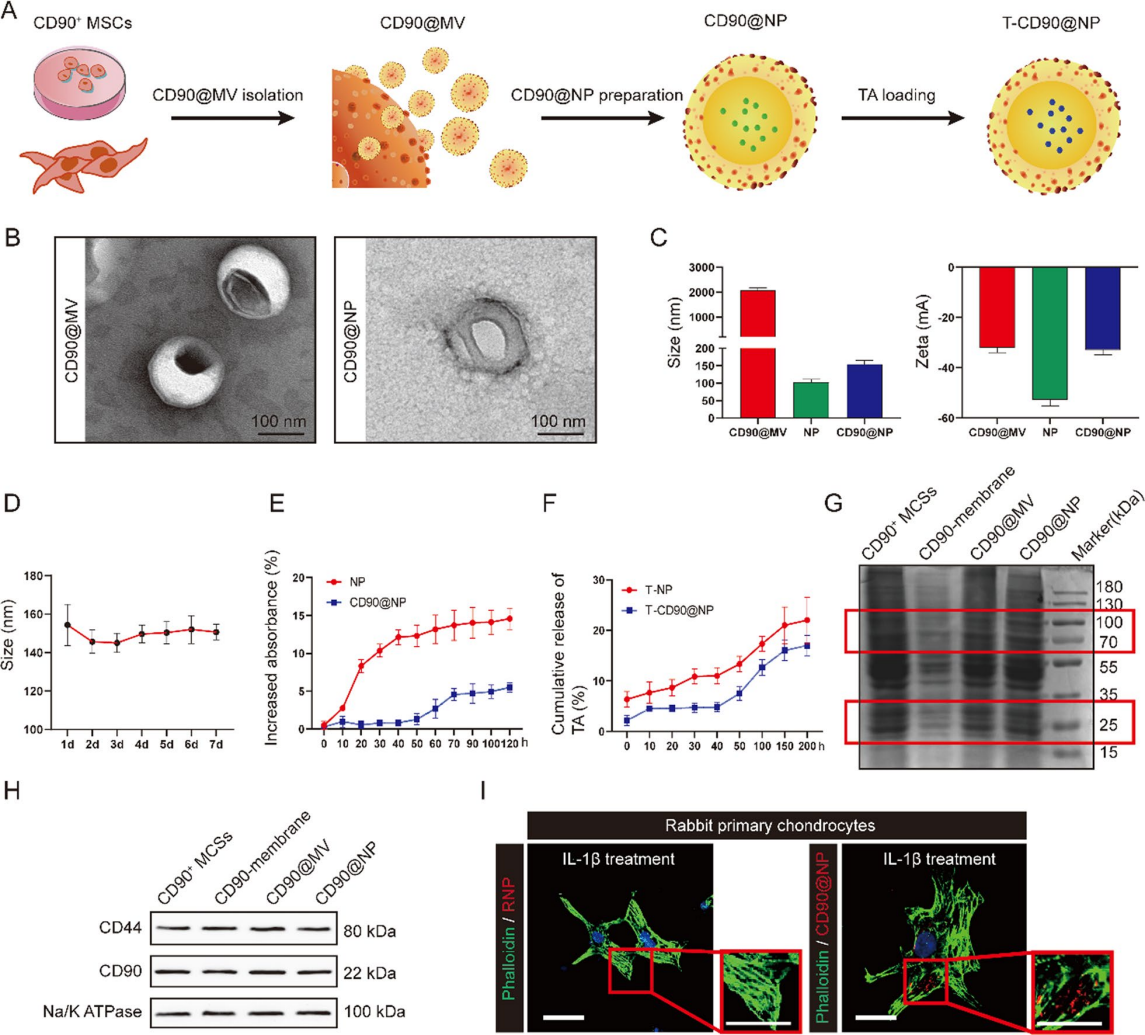
Characterization of CD90@NP. **A** Pattern diagram. **B** Transmission electron microscopy (TEM) images of CD90@MV and CD90@NP; Bar = 100 nm. **C** Size distribution and ζ potential of CD90@MV, NP, and CD90@NP; n = 3. **D** Change of CD90@NP size in PBS during 1 week; n = 3. **E** CD90@NP turbidity in serum; n = 3. **F** Release profile of TA encapsulated in NP and CD90@NP; n = 3. **G** Protein composition of CD90^+^ MSCs, CD90^+^ MSCs membranes, CD90@MV, and CD90@NP, analyzed by SDS-PAGE. **G**, **H** Western blot of CD44, CD90 and Na/K ATPase in CD90^+^ MSCs, CD90^+^ MSCs membranes, CD90@MV, and CD90@NP. **I** In vitro binding of CD90@NP (red, DID) and RNP (red, DID) to damaged primary chondrocytes (green); Bar = 100 μm, n = 3. *P < 0.05, **P < 0.01

Transmission electron microscopy (TEM) was used to observe the structure of the CD90@NP, which consisted of CD90@MV-wrapped NP, prepared for a DDS. The TEM results showed that, compared to CD90@MV, the CD90@NP had a significantly clear core and shell structure, indicating biofilm coating on the NP surface. (Fig. 3B). The size of the CD90@NP was approximately 154.3 ± 7.5 nm (Fig. 3C), slightly larger than the bar NP (103.3 ± 6.7 nm). We tested the zeta potential (ζ) of the CD90@NP. The zeta potential is the potential of the shear surface, an important indicator of biofilm stability. The results showed that the CD90@NP ζ potential (approximately − 33.1 ± 1.6 mV) was similar to that of the CD90@MV (natural cell membrane, approximately − 52.67 ± 1.5 mV). The CD90@NP was stable within 1 week with little change in size (Fig. 3D). CD90@MV had good biocompatibility because it is derived from CD90^+^ MSCs in the synovium. However, NP has poor biocompatibility and causes blood clotting. Thus, CD90NP biocompatibility was tested against that of CD90MV to investigate CD90@NP stability in the blood to reduce NP-caused blood clotting. Briefly, CD90@NP and NP were incubated with fetal bovine serum (FBS), followed by the detection of CD90@NP and NP coagulation. Coagulation was detected by testing turbidity changes over time. The coagulation results showed that NP had higher opacity (560 nm) for 20 min compared with CD90@NP, while the CD90@NP group remained stable within 120 h (Fig. 3E). The good biocompatibility and stability of CD90@NP may be related to the CD90@MV wrapped on the surface of the CD90NP, acting as a shield.

Triamcinolone acetonide-loaded CD90@MV (T-CD90@NP) was prepared as follow. In brief, T-CD90@NP preparation was optimized by inputting TA in the PLGA core (10% w/w). The TA release from T-CD90@NP was tested within 200 h to determine the TA capacity of the CD90@NP. The TA release rate from T-CD90@NP decreased compared to T-NP, which might be related to the CD90@MV covering the surface of the T-CD90@NP (Fig. 3F). The low TA release rate is beneficial for prolonging TA efficacy in joint cavity treatment.

The protein maintained on the CD90@NP surface (from CD90@MV) is essential for its biological function, such as cartilage repair. Thus, the CD90^+^ MSCs, CD90@MV, and CD90@NP protein composition were analyzed using sodium dodecyl sulfate-polyacrylamide gel electrophoresis (SDS-PAGE). The SDS-PAGE results showed that CD90^+^ MSCs and membrane of CD90^+^ MSCs, CD90@MV, and CD90@NP had similar protein compositions (both 80 Da and 22 kDa bands were similar). Thus, important protein bands (in the red-dotted frame) such as CD90 (22 kDa) and CD44 (80 kDa) were found (Fig. 3G). CD90^+^ MSCs and the membrane of CD90^+^ MSCs, CD90@MV, and CD90@NP were subjected to Western blotting to further analyze the two potential functional proteins, CD90 and CD44. CD90^+^ MSCs and the membrane of CD90^+^ MSCs, CD90@MV, and CD90@NP maintained similar expressions of CD44 and CD90 (Fig. 3H).

**Fig. 4:**
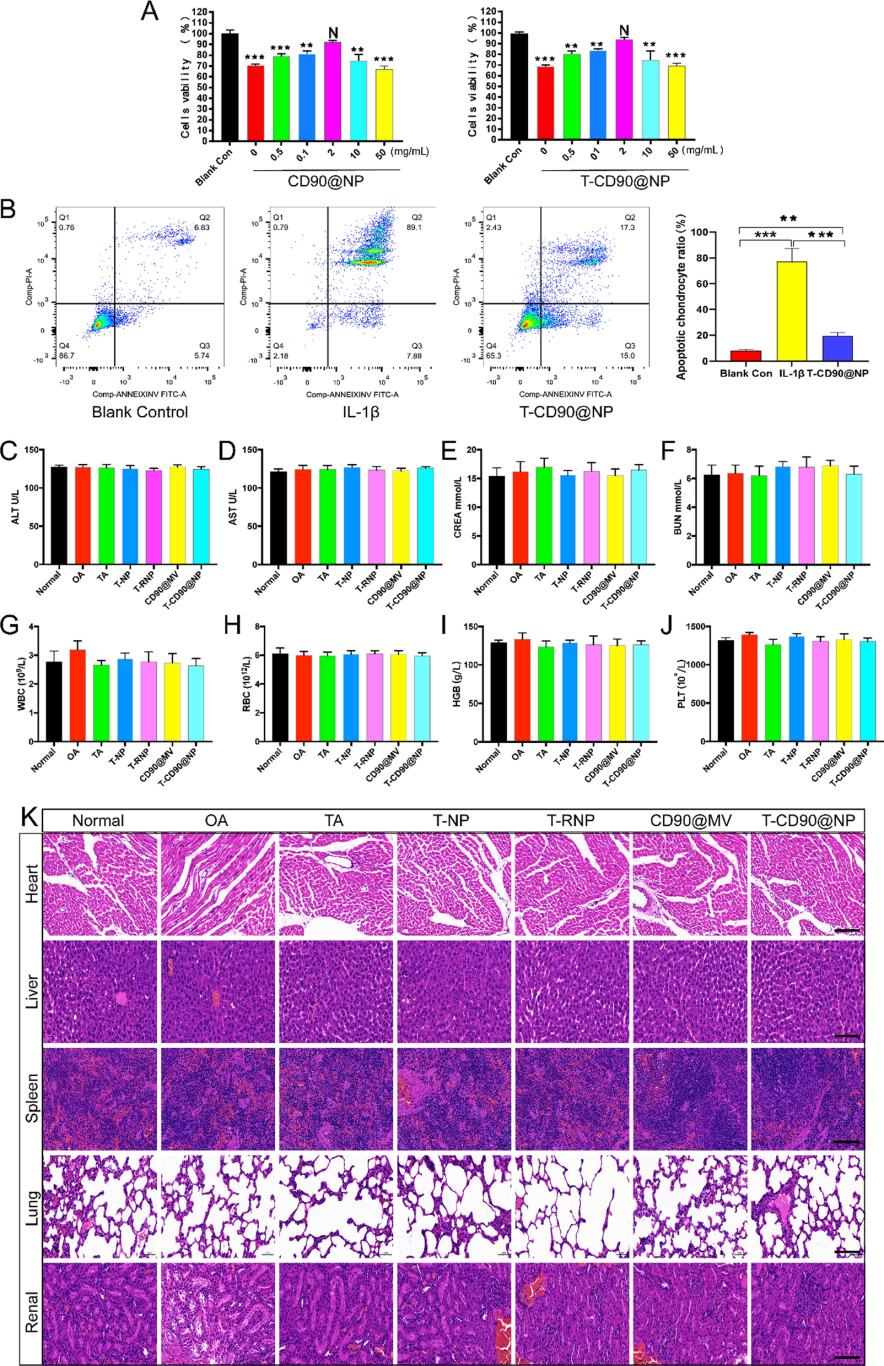
Preliminary toxicity study in vivo and in vitro. **A** MTT assays of CD90@NP and T-CD90@NP in primary chondrocytes; n = 3. **B** Flow cytometry assays of annexin V and propidium iodide (PI) in T-CD90@NP and IL-1βtreated primary chondrocytes; n = 3. **C**–**J** ELISA analysis of alanine aminotransferase (ALT), aspartate aminotransferase (AST), creatinine (CREA), blood urea nitrogen (BUN), white blood cell (WBC) count, red blood cell (RBC) count, hemoglobin (HGB) and platelet (PLT) levels in different groups; n = 3. **P < 0.01, ***P < 0.001, N = no statistical differences. **K** H&E staining of the heart, liver, spleen, lung and renal tissue in the different groups; Bar = 100 μm, n = 3

We constructed a physical damage model of chondrocytes (scratch model) and used DID (red) to stain T-CD90@NP and T-RNP and verify whether the damaged primary chondrocytes would take up the CD90@NP. T-CD90@NP uptake by the damaged primary chondrocytes was more than that of the T-RNP group, probably because CD90@NP inherits the properties of the CD90^+^ MSCs extracellular vesicles (Fig. 3I). Several assays were conducted to test T-CD90@NP cytotoxicity, including 3-(4,5-dimethylthiazol-2-yl)-2,5-diphenyltetrazolium bromide (MTT) assays in vitro, hepatotoxicity, routine blood tests and H&E staining of important organs. The MTT assay results showed that interleukin (IL-1β) treatment decreased the viability of primary chondrocytes, and the cell activities of T-CD90@NP and CD90@NP was the highest at a concentration of 2 mg/mL (Fig. 4A). Similarly, the apoptosis-related flow cytometry assay results showed that T-CD90@NP (2 mg/mL) could effectively decreased the apoptosis of primary chondrocytes (Fig. 4B). A serological enzyme-linked immunosorbent assay (ELISA) test was used to observe the effect of T-CD90@NP on liver function, kidney function and the circulatory system of rats. The results showed no abnormalities in alanine aminotransferase (ALT), aspartate aminotransferase (AST), creatinine (CREA), urea nitrogen (BUN), white blood cell (WBC) count, red blood cell (RBC) count, hemoglobin (HGB) and platelet (PLT) levels (Fig. 4C–J). Similarly, the H&E staining of the heart, liver, spleen, lung and kidney in all groups was normal (Fig. 4K).

### T-CD90@NP promote articular cartilage repair in the OA model rabbit

**Fig. 5:**
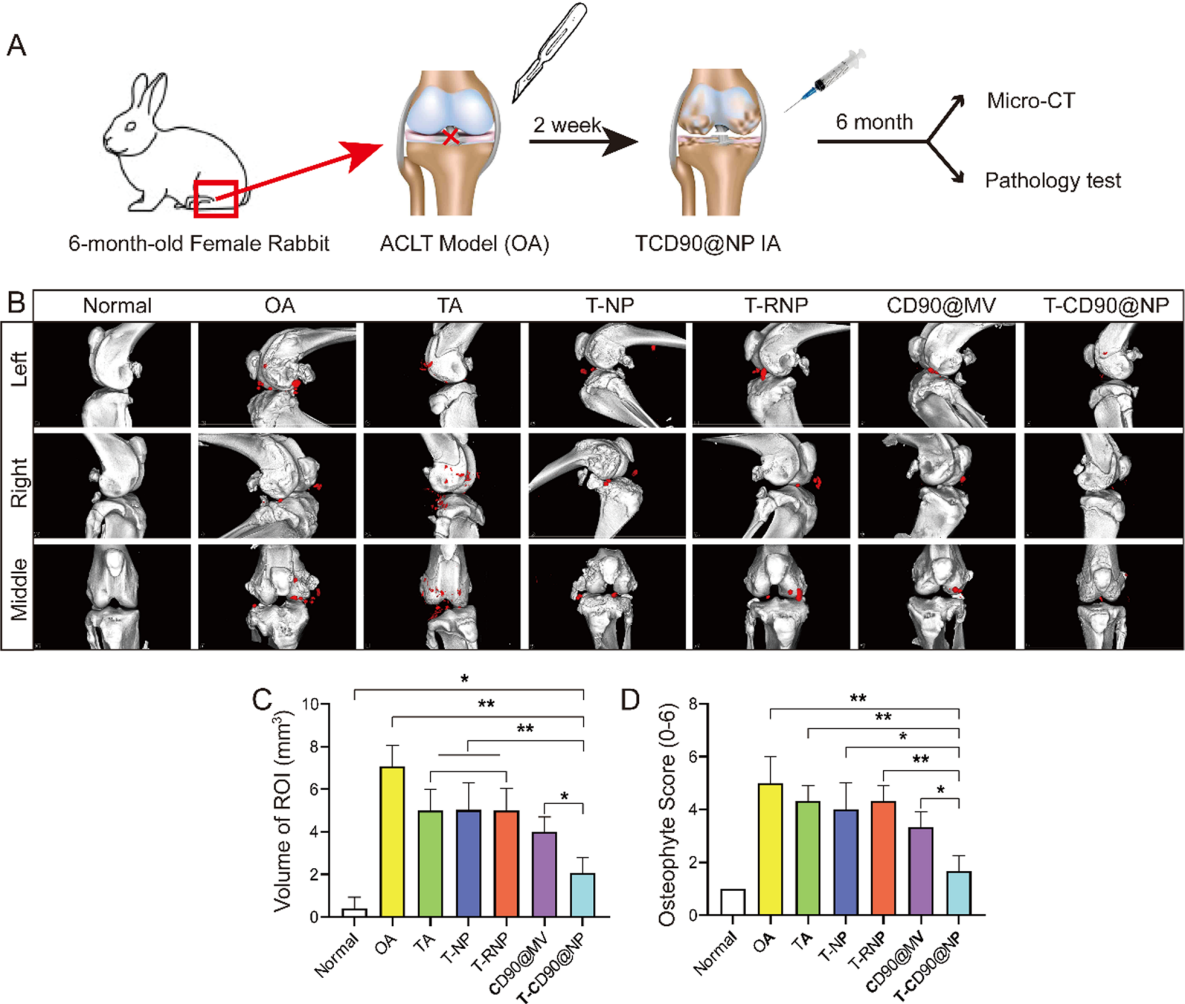
Micro-CT scan of the rabbit knee joint. **A** Pattern diagram. **B** Micro-CT of the knee joint in the different groups; n = 3. **C** Statistical analysis of the region of interest (ROI, red cubes marked osteophyte). **D** Statistical analysis of osteophyte scores; *P < 0.05, **P < 0.01

The study employed micro-CT to observe the formation of osteophytes in the knee joint cavity and subchondral bone defects in OA. The micro-CT results showed that 6 months after treatment, the subchondral bone surface in the T-CD90@NP group was smooth and similar to that of the normal group (Fig. 5B). However, the subchondral bone surface in the other groups was rough and displayed varying degrees of bone defects. Meanwhile, the subchondral bone in the OA and T-NP groups increasingly collapsed.

The severe damage induced numerous osteophytes, which appeared (red cubes marked osteophyte, region of interest, ROI) in each group of joint cavities in OA. Similarly, the volume of osteophytes in the T-CD90@NP group was significantly decreased compared to the other treatment groups (Fig. 5C). Likewise, the T-CD90@NP group showed lower osteophyte scores than the other groups (Fig. 5D). Altogether, these findings indicated that at 6 months, T-CD90@NP was beneficial for subchondral bone and osteophyte formation in the OA model rabbit.

**Fig. 6:**
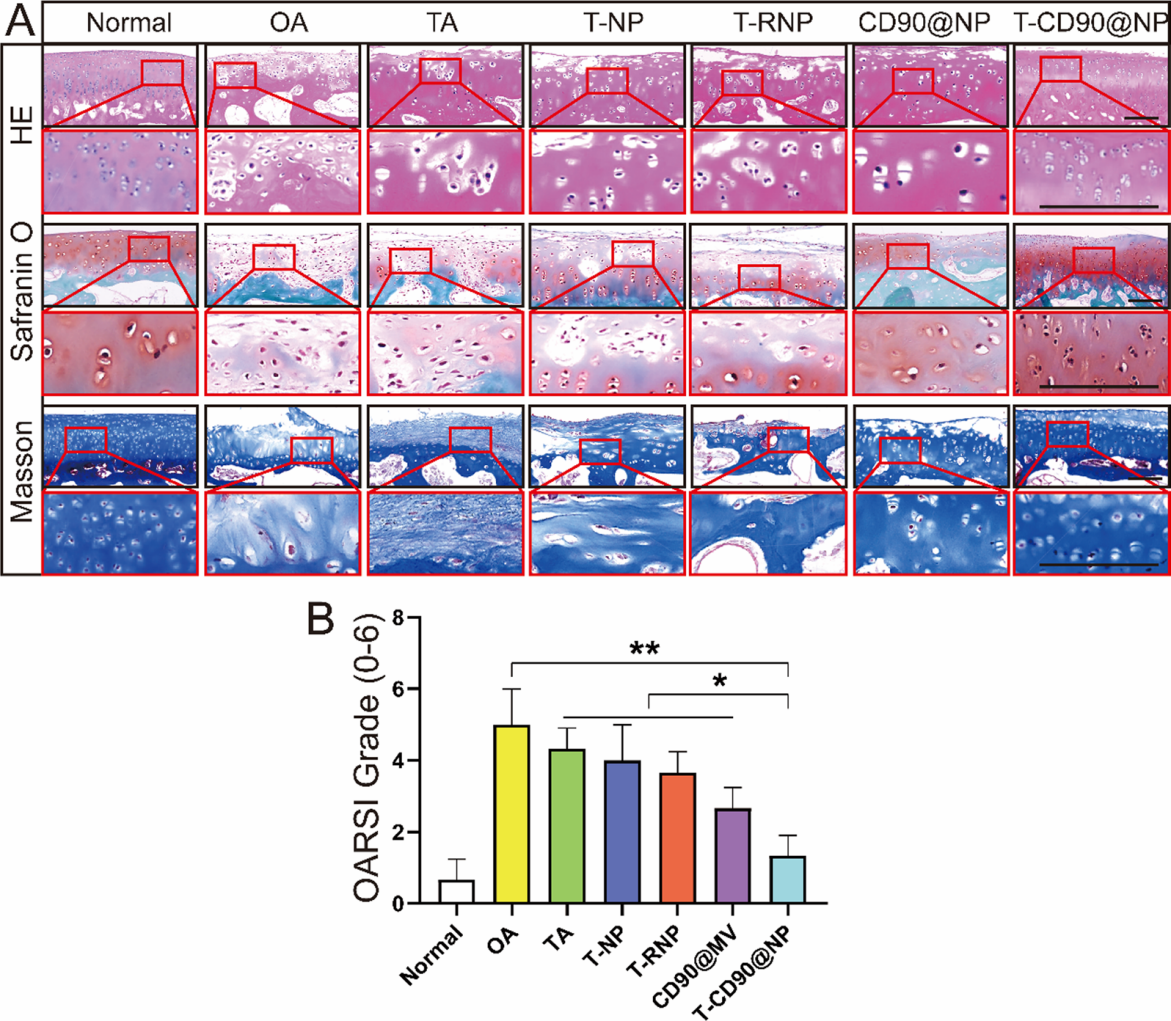
Pathological examination of the rabbit knee joint. **A** H&E, Safranin O, and Masson staining of knee cartilage in the different groups. Bar = 100 μm, n = 3. **B** Statistical analysis based on Safranin O. *P < 0.05, **P < 0.01, ***P < 0.001

The most damaged femoral trochlear cartilage was selected for pathological examination to observe the changes in cartilage structure 6 months after treating the OA model rabbit. The H&E staining and micro-CT results were similar. The chondrocytes in repaired T-CD90@NP tissue were neatly arranged, similar to the normal group. However, the repaired cartilage appeared as disorderly, fibrous, and loose tissue in the other treatment groups. The Safranin O fast green results showed that the T-CD90@NP and normal groups had the best cartilage repair compared to the other treatment groups. Masson staining is specific for fibrous tissue. The Masson staining results showed that the other treatment groups had increased fibrous tissue, exhibiting a disordered tissue structure, compared to the T-CD90@NP group (Fig. 6A). Moreover, the OARSI scores (histomorphology scores) were consistent with the staining results. The OARSI scores in the T-CD90@NP group were significantly lower than the other treatment groups but were higher than those in the normal group (Fig. 6B).

### T-CD90@NP regulates joint cavity inflammation by promoting macrophage polarization to the M2 phenotype in OA model rats

**Fig. 7:**
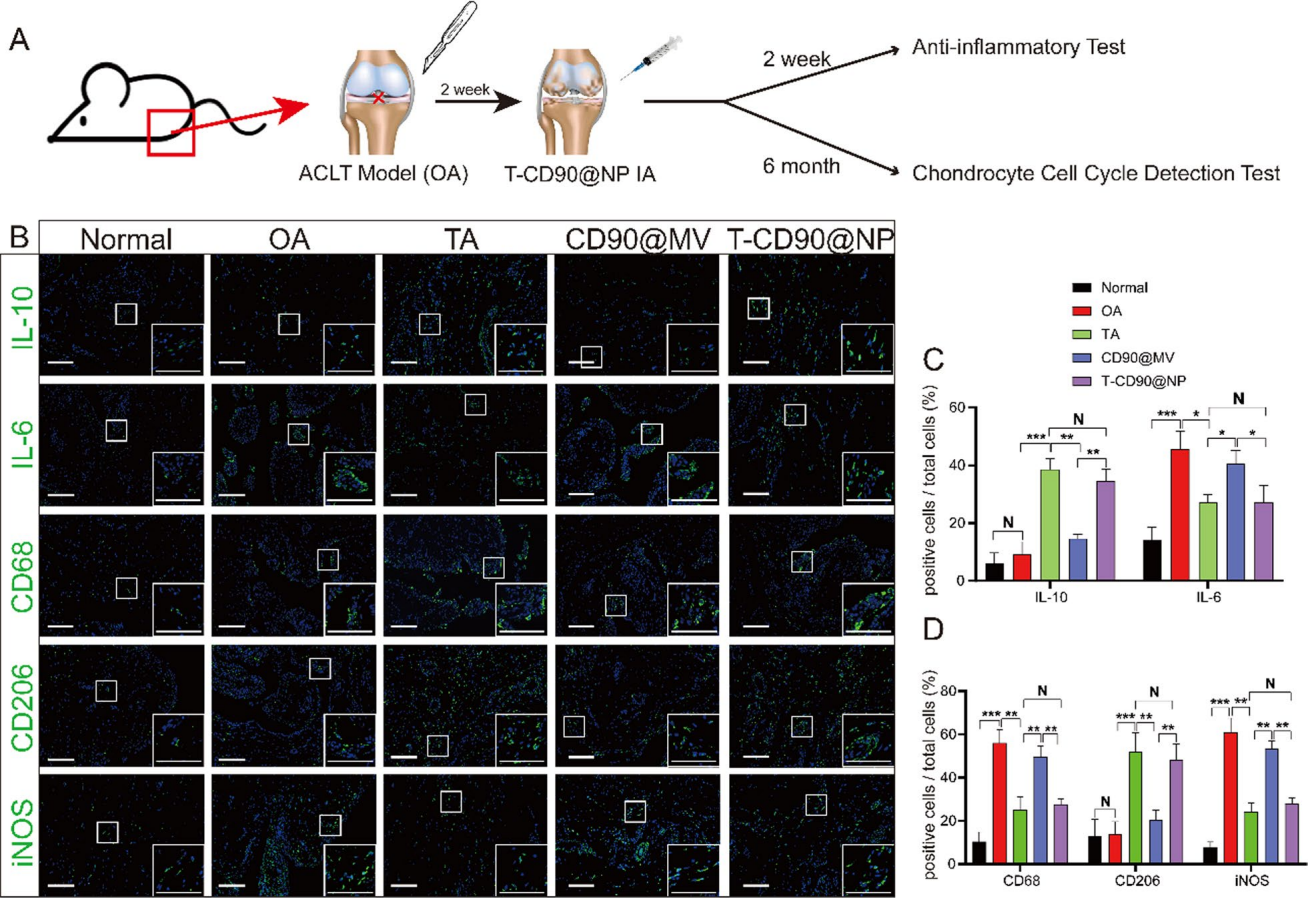
Detection of synovium inflammation levels in rats (**A**). Pattern diagram. **B** IL-10, IL-6, CD68, CD206, iNOS (green), and DAPI (blue) immunofluorescence staining of rat synovium in the different groups. Bar = 100 μm, n = 3. **C**, **D** Statistical analysis of immunofluorescence staining. *P < 0.05, **P < 0.01, ***P < 0.001

Regulating the inflammation of the joint cavity microenvironment after OA effectively promotes cartilage regeneration, and synovium macrophages regulate the inflammation levels in the microenvironment. We injected T-CD90@NP into the knee joint cavity in a rat OA model to verify whether the anti-inflammatory ability of T-CD90@NP was similar to that of TA treatment of OA. IF staining for the proinflammatory cytokine IL-6 and the anti-inflammatory cytokine IL-10 in the joint synovium reflected the inflammation level in the joint cavity microenvironment (Fig. 7A). Two weeks after treatment, the IL-10 expression in T-CD90@NP was significantly increased, similar to the TA group. However, the expression of IL-6 decreased compared to the other groups, a trend similar to the TA group. These results indicated that the T-CD90@NP and TA groups had similar anti-inflammatory abilities. Two weeks after OA induction, we performed IF staining for three macrophage polarization markers (CD68, CD206, and iNOS) to further explore the T-CD90@NP mechanism of regulating inflammation. Both IL-6 and IL-10 induced the polarization of macrophages (Fig. 7B). CD68 and iNOS significantly decreased in the T-CD90@NP and TA groups, whereas the expression of CD206 increased in the T-CD90@NP and TA groups compared to the other treatment groups (Fig. 7C). These results indicated that after 2 weeks of treatment, T-CD90@NP induced synovium macrophages in OA model rats to polarize to the M2 phenotype.

### T-CD90@NP promotes cartilage regeneration and reduces cartilage apoptosis in OA model rats

**Fig. 8:**
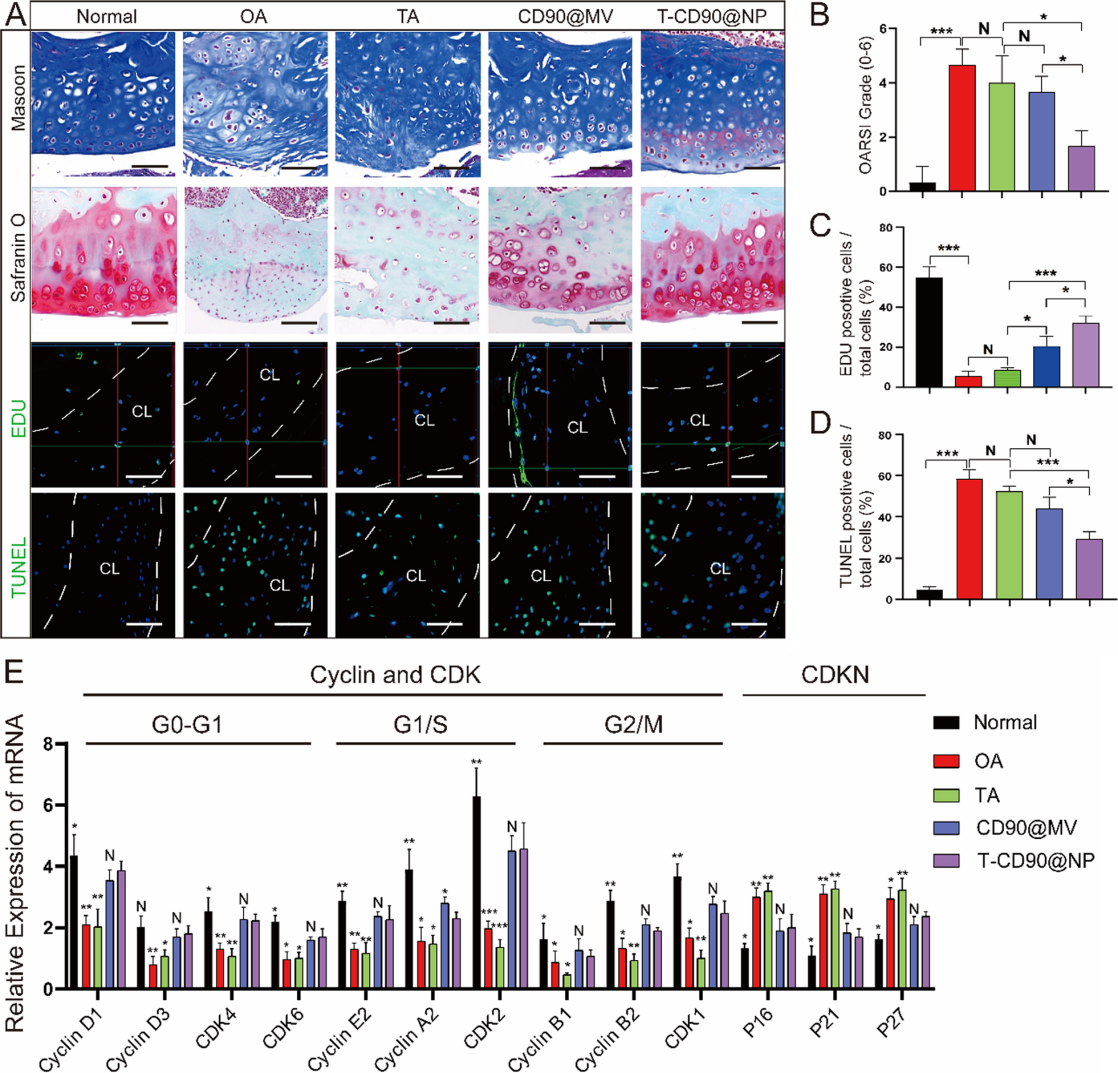
Regeneration and apoptosis of knee articular cartilage in rats. **A** Masson and Safranin O staining, and EDU, TUNEL (green), DAPI (blue) immunofluorescence staining of rat knee cartilage in the different groups. The region between the white dotted lines is the cartilage layer (CL). Bar = 100 μm, n = 3. **B**–**D** Statistical analysis of staining. **E** QPCR detection of cell cycle-related factors. *P < 0.05, **P < 0.01, ***P < 0.001, N = no statistical difference

Cartilage damage and apoptosis caused by OA are key yet difficult points in treatment. Therefore, methods to reduce cartilage apoptosis and promote cartilage regeneration need attention. We injected T-CD90@NP into the knee joint cavity of a rat OA model to detect cartilage regeneration and apoptosis after T-CD90@NP treatment. Six months after OA induction, we observed cartilage apoptosis and the cell cycle by IF and quantitative real-time polymerase chain reaction (qPCR). First, compared to the other groups, T-CD90@NPs repaired the cartilage layer and restored the ECM of the cartilage layer better (Fig. 8A, B). The cell regeneration marker (EDU) was enhanced in the cartilage layer of the T-CD90@NP and CD90@MV groups compared to the other treatment groups. There was little difference in EDU expression between the T-CD90@NP and CD90@MV groups (Fig. 8C).

However, cell apoptosis marker (TUNEL) expression was decreased in the T-CD90@NP group than in the other treatment groups. There was a decrease in the T-CD90@NP group compared to the CD90@MV group, probably due to the early anti-inflammatory ability of the enclosed TA (Fig. 8D). QPCR detected key cell cycle factors to explore the impact of T-CD90@NP on the chondrocyte cell cycle. Cell cycle promoters (cyclin and CDK family) were significantly increased in the T-CD90@NP and CD90@MV groups compared to the other groups. However, it was decreased by a cell cycle inhibitor (CDKN family) in the T-CD90@NP compared to the other treatment groups, similar to the CD90@MV group (Fig. 8E). The ability of T-CD90@NP to promote cartilage cell regeneration may be related to the CD90@MV surface cover because synovial mesenchymal stem cells produce CD90@MV, which promotes cartilage proliferation.

**Fig. 9:**
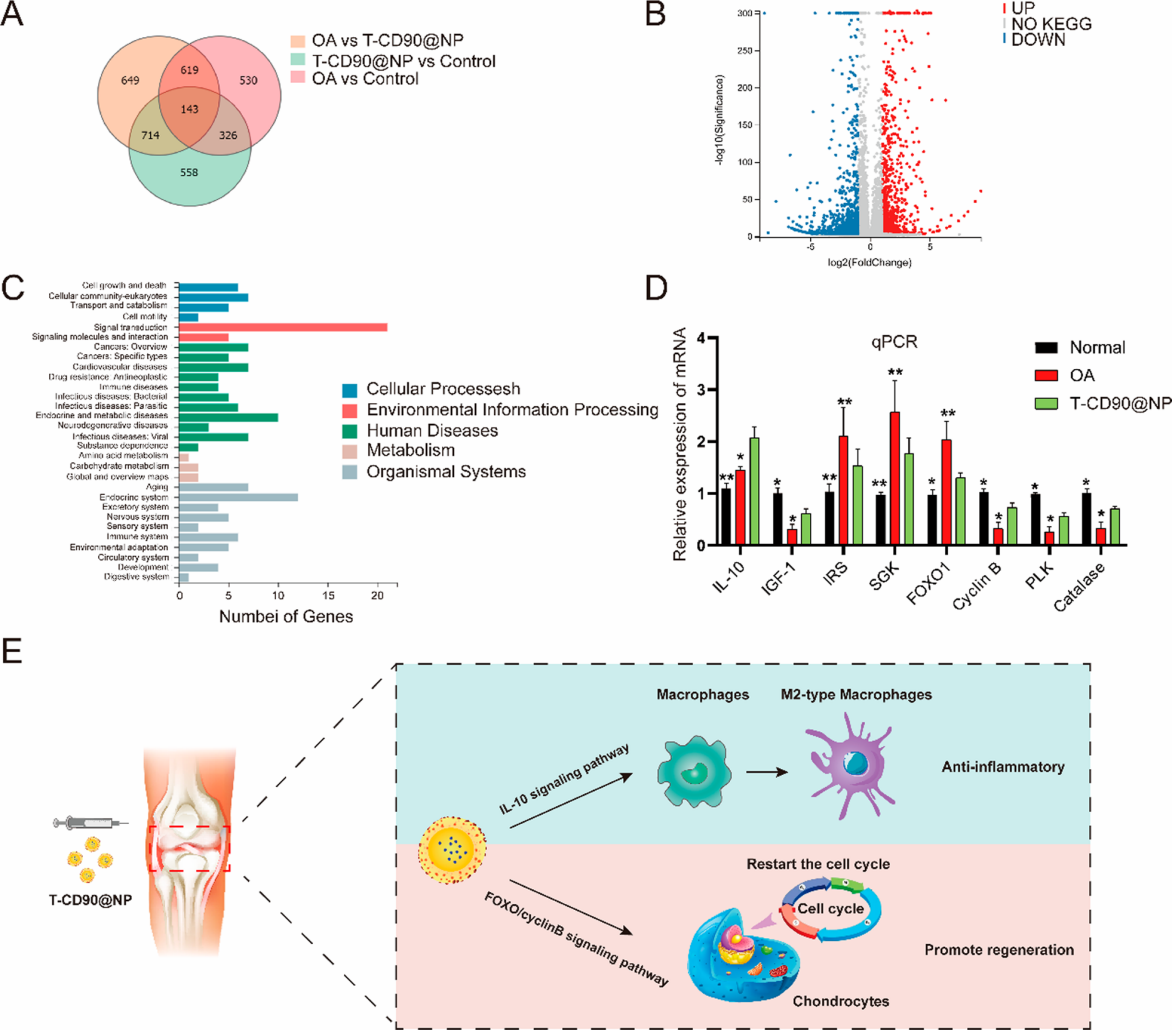
Detection of T-CD90@NP treatment effects (**A**). Venn diagram of mRNA sequencing. **B** Volcano charts of the OA and T-CD90@NP group comparison. **C** GO analysis of the OA and T-CD90@NP group comparison. **D** QPCR detection of the enriched FOXO signaling pathway-related factors; n = 3, *P < 0.05, **P < 0.01. **E** Schematic diagram of the T-CD90@NP cartilage repair mechanism after OA

Next, we used mRNA sequencing and bioinformatic analysis to explore the molecular mechanism of T-CD90@NP in promoting cartilage proliferation and anti-inflammation in rats 6 months after OA. T-CD90@NP substantially changed the expression of many genes in the cartilage of rats 6 months after OA (Fig. 9A, B). KEGG pathway analysis showed that the top 100 high-abundant mRNAs were enriched for cellular processes and environmental information (Fig. 9C). The study focused on the genes for cell growth and death, considering the T-CD90@NP biological test results. Indeed, the FOXO signaling pathway was enriched in T-CD90@NP-treated cartilage after OA (Additional file 1: Fig. S1). Both the qPCR and mRNA results showed that the IL-10, IGF1, cyclin B, PLK, and catalase expression increased, whereas that of IRS, SGK, and FOXO1 decreased in the T-CD90@NP group compared to the OA group (Fig. 9D). The other KEGG enriched pathways included the JAK-STAT, insulin, PI3K-Akt, and FOXO signaling pathways. In summary, these enriched pathways possibly regulate cartilage regeneration (Fig. 9E).

## Materials and methods

### Human cartilage and synovium

Human OA cartilage and synovium (OA group) were obtained from patients undergoing total knee replacement surgery (n = 5; aged 61.00 ± 3.71 years; one male and four females). The TA group included the human OA cartilage and synovium group that had received TA injections in the joint cavity. The samples were obtained from patients undergoing total knee replacement surgery (n = 5; aged 56.00 ± 4.21 years; two males and three females; received 3–4 TA intra-articular injections). Normal control cartilage and synovium (normal group) were obtained from patients who were involved in traffic accidents, with no history of arthritic disease (n = 4; aged 34.17 ± 7.32 years; two males and two females). The human cartilage was stained with H&E, Masson, Safranin O, IH (P21, P16, MMP13, and SOX9), and IF analysis (Ki67/DAPI). IF staining of the human synovium was (CD90/DAPI) also performed. The patients provided written consent, and the Ethics Committee of the First Affiliated Hospital of Jinzhou Medical University (Jinzhou, China) approved the study before the human tissue samples were harvested.

### Isolation and culture of CD90^+^ MSCs

Fresh knee synovium was obtained from humans (who had been involved in traffic accidents with no history of arthritic disease). The synovium was cut into 1-mm^3^ pieces under aseptic conditions and digested in 4 mg/mL type I collagenase (prepared in Dulbecco’s modified Eagle medium (DMEM). After 120 min of digestion at 37 °C, the synovium was filtered and centrifuged to obtain a synovial single-cell suspension. A culture medium containing 10% FBS (prepared in DMEM) was added to the culture flask and cultured at 37 °C.

### Flow cytometry

Single cells were resuspended and incubated with flow cytometry antibodies (CD90/CD45) at 4 °C for 30 min. CD90+CD45− cells were sorted using the BD Influx cell sorter (BD Biosciences, NJ, USA). The isolated cells were cultured with DMEM/F12 (Gibco, TX, USA) supplemented with 10% FBS (Gibco, TX, USA) and 100 IU/mL penicillin/streptomycin (Invitrogen, MA, USA) at 37 °C in a 5% CO_2_ atmosphere. Cells were passaged when they reached 90% density.

Cells at passage five were used to evaluate stem cell surface markers by flow cytometry. All cultured cells were digested into single cells with trypsin and resuspended in phosphate-buffered saline (PBS) with the appropriate antibodies (CD44/CD106). After incubation at 4 °C for 30 min, the cells were washed thrice with PBS and analyzed (Influx flow cytometer).

Next, all cultured cells were digested into single cells and resuspended in PBS with appropriate antibodies (PI/Annexin V). After incubation at 4 °C for 30 min, the cells were washed thrice with PBS and analyzed (Influx flow cytometer).

The flow antibodies used in this study were CD90-PECY7 (dilution 1:200), CD45-FITC (dilution 1:200), CD44-PE (dilution 1:200), CD106-APC (dilution 1:200), annexin V, and fluorescein isothiocyanate (FITC; dilution 1:200) (BD Biosciences).

### Viable cell count

The Cell Insight HCS and HCA systems (Thermo Fisher Scientific, MA, USA) were used to detect changes in the number of nuclei by nuclear staining (DAPI immunofluorescence staining) of CD90^+^ MSCs cells at the fifth (P5), tenth (P10), and fifteenth (P15) generations of culture.

### CD90^+^ MSCs differentiation ability

Adipogenic (HUXUC-90031), osteogenic (HUXUC-90021), and chondrogenic (HUXUC-90041) induction media (Cyagen Biosciences, CA, USA) were used to evaluate the ability of CD90^+^ MSCs to differentiate into osteocytes, adipocytes, and chondrocytes. After 21 days, Alcian blue, Alizarin red, and Oil red O staining detected the differentiation ability of CD90^+^ MSCs.

### Immunofluorescence and immunohistochemical staining

The cartilage of human and rabbit knee joints was decalcified using EDTA. The softened cartilage tissue and fresh synovium were washed with low-temperature PBS (4 °C), embedded in paraffin, and sectioned (2 μm). After deparaffinization and antigen retrieval, the obtained paraffin sections were blocked with 10% goat serum for 2 h at room temperature. For IF staining, the following antibodies were used: anti-Ki67 (ab238020, 1:800), anti-phalloidin (8878, 1:20), anti-DAPI (ab104139, 1:1), anti-IL-10 (ab9969, 1:1000), anti-IL-6 (ab259341, 1:1000), anti-CD68 (ab125212, 1:500), anti-CD206 (ab64693, 1:1000), and anti-iNOS (ab178945, 1:800) from Abcam (Cambridge, UK), and anti-CD90 (sc-53116, 1:1000) from Santa Cruz Biotechnology Inc. (TX, USA), as well as EDU (5-ethynyl-2′-deoxyuridine, E10187, 1 mg for intra-articular injection, once a week) (Thermo Fisher Scientific, MA, USA), and TUNEL staining (11684817910, 1:300) (Roche, Basel, Switzerland).

For IH staining, the following antibodies were used: anti-SOX9 (EPR14335, 1:500), anti-MMP13 (EPR21778, 1:1000), anti-P21 (HUGO291, 1:500), and anti-P16 (ab151303, 1:500), as well as Fontana-Masson stain (ab150669) from Abcam (Cambridge, UK), Safranin O Fast Green Stain, and H&E stain from Servicebio (Wuhan, China). Photographic images were obtained after incubation with the appropriate secondary antibodies using the LCM800 microscope (Zeiss, Jena, Germany).

### Animal experiment

This Institutional Animal Care of Jinzhou Medical University approved the study protocol. Thirty-five New Zealand white rabbits (6-month old males) and 42 adult SD rats (6-months old males) were purchased from and housed at the experimental animal center of Jinzhou Medical University. The rabbits were randomly divided into seven groups of five rabbits each. The groups included normal (the skin was cut without damaging the ligaments), OA (rabbits underwent anterior cruciate ligament transection (ACLT) surgery to injure the joint, and the OA model was successfully induced after 2 weeks), TA (the OA model + 12 mg TA, IA once a week for 24 weeks), T-NP (OA model + NP wrapped TA, 12 mg IA once a week for 24 weeks), T-RNP (OA model + RMV wrapped TA, 12 mg once a week for 24 weeks), CD90@MV (OA model + CD90@MV, 12 mg IA once a week for 24 weeks), and T-CD90@NP (OA model + T-CD90@NP, 12 mg IA once a week for 24 weeks).

Subsequently, the rats were randomly divided into seven groups, with six randomly assigned rats per group. The groups included: normal (the skin was cut without damaging the ligaments), OA (rats underwent anterior cruciate ligament transection (ACLT) surgery to injure the joint, and the OA model was successfully induced after 2 weeks), TA (OA model + 0.25 mg TA, IA once a week), T-NP group (OA model + NP wrapped TA, 0.25 mg IA once a week), T-RNP (OA model + RMV wrapped TA, 0.25 mg IA once a week), CD90@MV (OA model + CD90@MV, 0.25 mg IA once a week), and T-CD90@NP (OA model + T-CD90@NP, 0.25 mg IA once a week). After 2 weeks of treatment, three rats in each group were randomly selected for synovial tests, and the other three were used for knee joints tests. After inducing the OA model, the rabbits and rats were allowed to move and eat freely.

### Preparation of CD90@MV, CD90@NP, T-NP, T-RNP, and T-CD90@NP

#### CD90@MV

CD90^+^MSCs were taken from human synovium (normal group) by fluorescence-activated cell sorting and cultured in DMED supplemented with 10% FBS. The CD90^+^ MSCs were washed thrice with PBS and subsequently incubated in 5 mL FBS-free DMEM with 10 μg/mL CB (Abcam, Cambridge, UK) for 2 h at 37 °C to obtain CD90@MV. The CD90@MV and cells were detached from the cell culture flask using 5 mL of DMEM and vortexed for 8 min at 37 °C to isolate CD90@MV. Subsequently, 5 mL of FBS was added to the tube to obtain a final concentration of 50% FBS. The mixture was centrifuged at 3000 rpm for 5 min to remove the cells, impurities, and large CD90@MV aggregates. Next, the obtained supernatant was centrifuged at 8000 rpm for 10 min to get CD90@MV. The obtained CD90@MV was washed thrice with 0.25% EDTA (diluted in PBS) to remove the nucleus and cytoplasm. The CD90@MV protein quantity was determined using the BCA assay (Thermo Fisher Scientific, MA, USA).

#### CD90@NP

The CD90@MV membrane-coated NP (CD90@NP) was prepared following a previous study [28]. Briefly, 0.7 dL/g carboxy-terminated 50:50 PLGA (LACTEL Absorbable Polymers, Birmingham, UK) was used to form the PLGA NP cores using a nanoprecipitation method. PLGA was dissolved in acetone solution (10 mg/mL), and 1 mL of the PLGA solution was added to 2 mL deionized water to remove the acetone solution via a vacuum. The resulting NP solution was mixed with CD90@MV at a ratio of 1:10 (w/w, protein: PLGA) and sonicated using a water bath sonicator (FPMRC-DCS-250H, Fuguang, China) for 5 min at 80 W.

#### T-NP, T-RNP, and T-CD90@NP

Red blood cell membranes were collected from healthy human and coated with nanoparticles (RNP) following previous methods [28]. The RNP and CD90@NP were labeled with DiD (5 μM, Fanbo Biochemicals Co. Ltd, Beijing, China) at the concentration of 0.2% (w/w) for 10 min. Triamcinolone acetonide (18026, Cayman Chemical Company, MI, USA)-loaded NP (T-NP), RNP (T-RNP), and CD90@NP (T-CD90@NP) were prepared by adding 20% (w/w) TA to the respective solutions during the PLGA core preparation.

The structure micro humid of the CD90@NP was observed using the HT7800 transmission electron microscope (Hitachi Ltd, Tokyo, Japan). The ζ potential (mA) and particle size (nm) of the CD90@MV, NP, RNP, and CD90@NP were detected by a Malvern Zetasizer Nano ZS90 nanoparticle size system (Malvern, UK). T-CD90@NP was collected via centrifugation at 12,000×*g* for 45 min and dissolved in dimethyl sulfoxide to determine the DLC and EE of TA. The TA content in T-CD90@NP was tested by ELISA (Abnova, Taipei, Taiwan). The following formula was used to calculate the DLC and EE:

$${\text{DCL}} \left( \% \right) = \frac{{{\text{TA}}\;{\text{encapsulated}}\;{\text{in}}\;{\text{T - CD}}90@{\text{NP}}}}{{{\text{weight}}\;{\text{of}}\;{\text{T-CD}}90@{\text{NP}}}} \times 100\% ,$$

$${\text{EE }}\left( \% \right) = \frac{{{\text{TA}}\;{\text{encapsulated}}\;{\text{in}}\;{\text{T-CD}}90@{\text{NP}}}}{{{\text{total}}\;{\text{TA}}}} \times 100\% .$$

### Preparation and characterization of CD90@NP

Changes in CD90@NP sizes were detected for 7 days at 4 °C to evaluate the stability of the CD90@NP. CD90@NP was incubated with 50% FBS (500-S, AusgeneX, Queensland, Australia), and the turbidity over time was analyzed at 560 nm using a microplate reader to evaluate the stability of the CD@90NP in blood [29]. The rate of TA release from the T-CD90@NP was determined by adding 2 mg of T-CD90@NP to 1 mL of 0.5% (v/v) Tween 80 solution (diluted in DMEM) in dialysis bags, then centrifuging after 10, 20, 30, 40, 50, 60, 70, 90, 100, and 120 h (n = 3) to obtain 1 mL of supernatant. After that, the amount of TA was measured by ELISA.

### CD90@NP protein composition

The CD90@NP protein composition was investigated by SDS-PAGE. Briefly, proteins extracted from the synovial MSCs, CD90^+^ MSC membranes, and CD90@MVs and CD90@NPs were adjusted to 2 mg after BCA protein concentration determination. After electrophoresis and transfer, the protein was stained with Coomassie Brilliant Blue and imaged. Similarly, anti-CD90 (ab225), anti-CD44 (ab9524) and Na/K ATPase (ab167390; Abcam (Cambridge, UK) reactions were detected and imaged.

### In vitro binding of CD90@NP

We conducted an in vitro phagocytosis experiment of CD90@NP by primary chondrocytes to determine whether chondrocytes could phagocytose CD90@NPs. Briefly, the extracted primary chondrocytes from suckling rabbits were inoculated onto 24-well plates. IL-1β (10 μg/L, Peprotech, USA) was used for cell damage (treated 24 h at 37 °C). Next, DiD-labeled CD90@NPs were added to the chondrocyte cultures for 8 h at 4 °C and washed thrice with Hanks balanced salt solution (HBSS; Gibco, TX, USA). DiD-labeled RNP were used as the control group. After that, the absorbance at 633 nm (DiD) and 488 nm (phalloidin, labeled chondrocytes) was imaged using an LCM800 microscope.

### Cell viability assays

Primary chondrocytes were cultivated in DMEM and 10% FBS, after which MTT assays were conducted to evaluate cell viability. In brief, primary chondrocytes were treated with CD90@NP or T-CD90@NP (0, 0.5, 2, 5, 10, and 50 mg/mL), then treated with IL-1β for 24 h at 37 °C. Subsequently, MTT (20 μM, Sigma- Aldrich, USA) was added to each well, and the plates were incubated for 4 h at 37 °C. Dimethyl sulfoxide (150 μL, Sigma-Aldrich) was added to each well and the absorbance at 490 nm was recorded.

### ELISA detection

After 10 min of natural coagulation at room temperature, rat blood (0.3 mL) was centrifuged for 20 min (3000 rpm), and the serum was collected for subsequent analyses. Next, ALT, AST, CREA, BUN, WBC count, RBC count, HGB, and PLT levels were detected by ELISA (GM1149, Servicebio, China), and the absorbance at 340 nm was recorded.

### Micro-CT detection

The knee joint of rabbits from the different groups was examined using a micro-CT imaging system (Siemens, Munich, Germany). The images were further reconstructed and analyzed using Siemens Multimodal 3D Visualization software (Siemens, Munich, Germany) to calculate the region of interest (ROI, red cubes marked osteophyte) volume.

### qPCR and RNA-sequence detection

RNA isolation and qPCR analysis were performed as described. Briefly, total RNA was extracted from cartilage tissues using TRIzol reagent (Invitrogen, MA, USA). RNA (1 mg) was reverse transcribed using the RevertAid First-Strand cDNA Synthesis Kit (Thermo Fisher Scientific, MA, USA) to obtain cDNA. The cDNA templates were analyzed using qPCR with the SYBR Green reagent (Roche, Basel, Switzerland) and pre-designed primers (Sangon Biotech, Shanghai, China).

The Ct values were normalized to that of GAPDH. The relative mRNA abundance was calculated using the ΔCt method [relative mRNA abundance = 2 − (Ct gene of interest − Ct GAPDH)]. The qPCR experiments were performed in triplicate. The rat primer sequences are listed in Table 1. For the RNA-seq experiments, isolated RNA samples were sent to Huada Gene Research Institute (Guangdong, China) for sequencing. The raw tag data were produced using the sequencing-by-synthesis method, and the institute processed the transcriptomic data.

**Table 1:** qPCR primers for key factors of rat chondrocyte cycle

Name	Gene name	Forward (5′ → 3′)	Reverse (5′ → 3′)
Gapdh	GAPDH	GCATCTTCTTGTGCAGTGCC	GATGGTGATGGGTTTCCCGT
Cyclin D1	CCND2	ACCTGTGAGGAAGCCATTCG	CCAGCGTGTCCCTTCTCATT
Cyclin D3	CCND3	ACACGCGTCGCTTCTCCTA	TGTGACATCTGTGGGAGTGC
CDK4	CDK4	ACCAGGATCTCCCACTAGCA	TCAGGTCCCGGTGAACAATG
CDK6	CDK6	GGCCGCAGTAGTCAGTTACC	CCAACACTCCAGAGGTCCAC
Cyclin E2	CCNE2	CGCAGTAGCCGTTTACAAGC	TCACTGCAAGCACCATCAGT
Cyclin A2	CCNA2	GTCAACCCCGAAAAAGTGGC	GCCTTCCATGTGTCTGACCAA
CDK2	CDK2	AGGCGGCAACATTGTTTCAA	GACAGGGACTCCAAAGGCTC
Cyclin B1	CCNB1	GGTCGATGTGGAGCAGCATA	GGCAAAATGCACCATGTCGT
Cyclin B2	CCNB2	TGGCTGGTCCAAGTCCATTC	TGTGCTGCATGACTTCCAGT
CDK1	CDK1	AGGACCAGCTCACAAAAGGG	GTGGAAAAGCGGCTTCTTGG
P16	CDKN2A	AACACTTTCGGTCGTACCCC	CTCCCTCCCTCTGCTAACCT
P21	CDKN1A	AAGCCCGAGTTCCTGCTAAC	ATCGGCGCTTGGAGTGATAG
P27	CDKN1B	GACTCACTCGCGGCTCC	TGTTTACGTCTGGCGTCGAA

### Statistical analysis

The data were analyzed using GraphPad Prism 8 (GraphPad Software Inc., CA, USA). Significant differences were evaluated using an unpaired Student’s t-test to compare two groups and one-way analysis of variance (ANOVA) for multiple group comparisons. The data are expressed as the mean ± standard deviation (SD), and a *P*-value of < 0.05 indicated a significant difference.

## Discussion

IA treatment with glucocorticoid promotes analgesia and reduces inflammation in OA [30]. However, its long-term use can lead to the loss of cartilage [31]. Thus, it is important to address the problem of the loss of cartilage content but retain anti-inflammatory and analgesic ability. Notably, the injection of synovial MSCs can effectively increase the chondrocyte content and promote cartilage regeneration in OA [32]. Thus, proposals to construct the complexes of synovial MSCs combined with glucocorticoids to incorporate anti-inflammatory, analgesic, and cartilage content-promoting abilities have been put forward. Some DDSs such as PEGylated liposomes/NPs have been designed based on their excellent drug loading and slow-release ability to address this challenge. Previous studies postulated that NPs had a good loading and a slow TA release capacity.

In this study, long-term IA of TA had limited therapeutic effects on the cartilage of patients with OA. This phenomenon was attributed to the arrest of the chondrocyte cycle, leading to the loss of cartilage content. This challenge was addressed by preparing NPs loaded with TA wrapped with synovial MSC membranes. CD90^+^ MSCs were extracted from human synovial tissue, and cytochalasin B was subsequently used to relax the interaction between the membrane and cytoskeleton of CD90^+^ MSCs to stimulate CD90@MV secretion. CD90^+^ MSCs were chosen because they are decreased in the synovium of OA patients under TA treatment and numerous reports have highlighted their importance in cartilage proliferation. The interaction between the membrane and cytoskeleton of CD90^+^ MSCs was subsequently weakened because the CD90^+^ MSCs -associated ability to promote cartilage proliferation and activate the cell cycle is significantly mediated by the membrane protein function, and thus cloaking of NP with bio-membranes to maintain the protein activity is mostly necessary. The traditional process of membrane isolation was not used because of its complexity and involves nuclear disposal, cell lysis, and centrifugation, which results in inefficiency and the loss of the membranes protein activity [33, 34]. The NP loaded with TA was then wrapped in CD90@MV to prepare T-CD90@NP. The CD90@NP exhibited great stability and the ability to release TA. Both CD90@MV and CD90@NP had similar protein compositions and were rich in CD90, inducing proliferation function, and CD44 proteins, inducing cell adhesion function [35]. These findings suggested that CD90@NP can perform protein functions like CD90^+^ MSCs and CD90@MV. Moreover, CD90@NP could be taken up in large quantities by primary chondrocytes than RNP (bar) after injury. Succinctly, the T-CD90@NP demonstrated good material and biological properties.

Rabbit OA animal models were used to verify the efficacy of T-CD90@NP, and rat OA animal models were used to explore the molecular mechanisms. TA administered via IA injection once a week for 24 weeks led to limited cartilage repair in the rabbit model. These findings were similar to those of the OA group. However, T-CD90@NP effectively repaired the cartilage tissue after OA. Recent studies postulated that short-term TA IA treatment effectively reduced the inflammation level in the joint cavity and had a great analgesic effect. In contrast, long-term TA IA treatment had a limited ability to repair cartilage tissue. The long-term effects of TA are attributed to the decrease in cartilage content. TA activates cell cycle inhibitors and inhibits cell cycle-activating factors, leading to cell cycle arrest. This cell cycle arrest deactivates chondrocytes and reduces the secretion of the cartilage matrix, thereby reducing the ability of cartilage tissue to repair [36]. The rat OA model was subsequently used to explore the mechanism of action of T-CD90@NP against cartilage loss caused by TA. Recent studies postulated that synovial inflammation significantly impacted cartilage repair and was closely related to OA development [37, 38]. M2-type macrophages reduce inflammation in the microenvironment of the synovial tissue by secreting IL-10, which induces cartilage repair [39]. Notably, T-CD90@NP reduced synovial inflammation and promoted the polarization of macrophages to the M2 type in the synovium during the early stages of treatment. Similar findings were observed upon TA treatment. The anti-inflammatory ability of T-CD90@NP was associated with the retention of TA wrapped in the NP.

Moreover, long-term treatment using T-CD90@NP effectively reduced chondrocyte apoptosis and increased their proliferation in the damaged area in the rat OA model. T-CD90@NP increased cell cycle promoting factors (cyclin and CDK family) but decreased the cell cycle inhibitor (CDKN family). Its ability to restart the cell cycle was attributed to the CD90@MV covered on its surface, which retained the ability of CD90^+^ MSCs to promote cartilage proliferation.

The study further explored the molecular mechanisms of T-CD90@NP in promoting chondrocyte proliferation and anti-inflammation. The genes differentially expressed upon T-CD90@NP treatment were found to be enriched in cell growth and death. These findings were based on the pro-proliferation and anti-inflammatory ability of T-CD90@NP. Notably, the differentially expressed genes were enriched in the FOXO signaling pathway, associated with varying biological phenomena, such as cell proliferation, apoptosis, and DNA damage caused by oxidative stress [40, 41]. T-CD90@NP was found to regulate the insulin, PI3K/AKT/FOXO, and Jak/STAT pathways. It can reduced the phosphorylation level of FOXO to increased cyclinB and PLK to restart the cell cycle and catalase to increased DNA repair. It can also increased IL-10, which is secreted by type 2 macrophages, consequently promoting the polarization of type 2 macrophages. Collectively, T-CD90@NP promoted the regeneration of chondrocytes and reduces apoptosis via the FOXO pathway. It also influenced the polarization of type 2 macrophages to regulate inflammation by increasing IL-10 secretion.

Currently, cell-derived MVs such as stem cells and tumor cells are considered valuable new tools for regenerative medicine. This is related to the fact that MVs are less immunogenic and have most of the biological activity of the parent cells. Interestingly, the MVs secreted by special cells have better cell targeting, which lays the foundation for the active identification of and treatment with MVs [42]. Moreover, based on the active recognition of cells by MVs, the MVs themselves can be loaded with substances such as drugs, DNA, or nanoparticles required for treatment to achieve the purpose of further treatment [43]. Therefore, MV-nanoparticles have great potential as a diagnosis and treatment platform in regenerative medicine.

## Conclusion

This study successfully used NP to combine CD90^+^ MSCs with TA (T-CD90@NP) to combat the cartilage loss caused by TA for OA treatment. The study demonstrated that CD90@NP is a promising material for mimicking MSCs and can be employed in drug loading. Notably, T-CD90@NP retained the ability to promote cartilage proliferation (CD90^+^ MSCs) and anti-inflammation (TA). This system provides a novel strategy for the clinical application of stem cells and glucocorticoids in OA to improve the therapeutic outcomes of patients. It also provides baseline information for future related studies.

## Supplementary Information

**Additional file 1: Figure S1.** KEGG analysis showed the enrichment of the FOXO signaling pathway in the OA and T-CD90@NP group comparison. Red represents increased expression, and green represents decreased expression.

## Data Availability

The data that supports the findings of this study are available from the corresponding author on request.

## Abbreviations

OA: Osteoarthritis
IA: Intra-articular
MSCs: Mesenchymal stem cells
NP: Nanoparticle
DDSs: Drug delivery systems
CD90@MV: CD90^+^ MCSs-derived microvesicle
CD90@NP: CD90^+^ MCSs-coated nanoparticle
CB: Cytochalasin B
PLGA: Poly(lactic-co-glycolic acid)
TA: Triamcinolone acetonide
T-CD90@NP: Triamcinolone acetonide encapsulated in CD90@NP
RNP: Erythrocyte membrane-coated nanoparticles
MMP13: Matrix metalloproteinases
ECM: Extracellular matrix of chondrocytes
SOX9: Chondrocyte differentiation and proliferation
MSCs: Mesenchymal stem cells
NP: Nanoparticle
DDSs: Drug delivery systems
CD90^+^ MSCs: Human synovial CD90-positive MSCs
CD90^+^@MV: Microvesicles extracted from CD90^+^ MSCs
T-CD90^+^@NP: CD90^+^@MV coated NPs with TA
DDS: Drug delivery system
OARSI: Osteoarthritis Research Society International
TEM: Transmission electron microscope
HCS: High content screening
HCA: High content analysis
ACLT: Anterior cruciate ligament transection
SD: Standard deviation
IF: Immunofluorescence
IH: Immunohistochemical
BCA: Bicinchoninic acid protein
PLGA: Poly (lactic-co-glycolic acid)
DID: 1,1′-Dioctadecyl-3,3,3′,3′-tetramethylindodicarbocyanine, 4-chlorobenzenesulfonate salt
DLC: Drug loading capacity
EE: Encapsulation efficiency
HBSS: Hank’s balanced salt solution
