# Supplementary figures and images for: Triamcinolone acetonide-loaded nanoparticles encapsulated by CD90+ MCSs-derived microvesicles drive anti-inflammatory properties and promote cartilage regeneration after osteoarthritis
Source: J Nanobiotechnology. 2022 Mar 19;20:150. doi: 10.1186/s12951-022-01367-z (PMC8934450; doi:10.1186/s12951-022-01367-z)

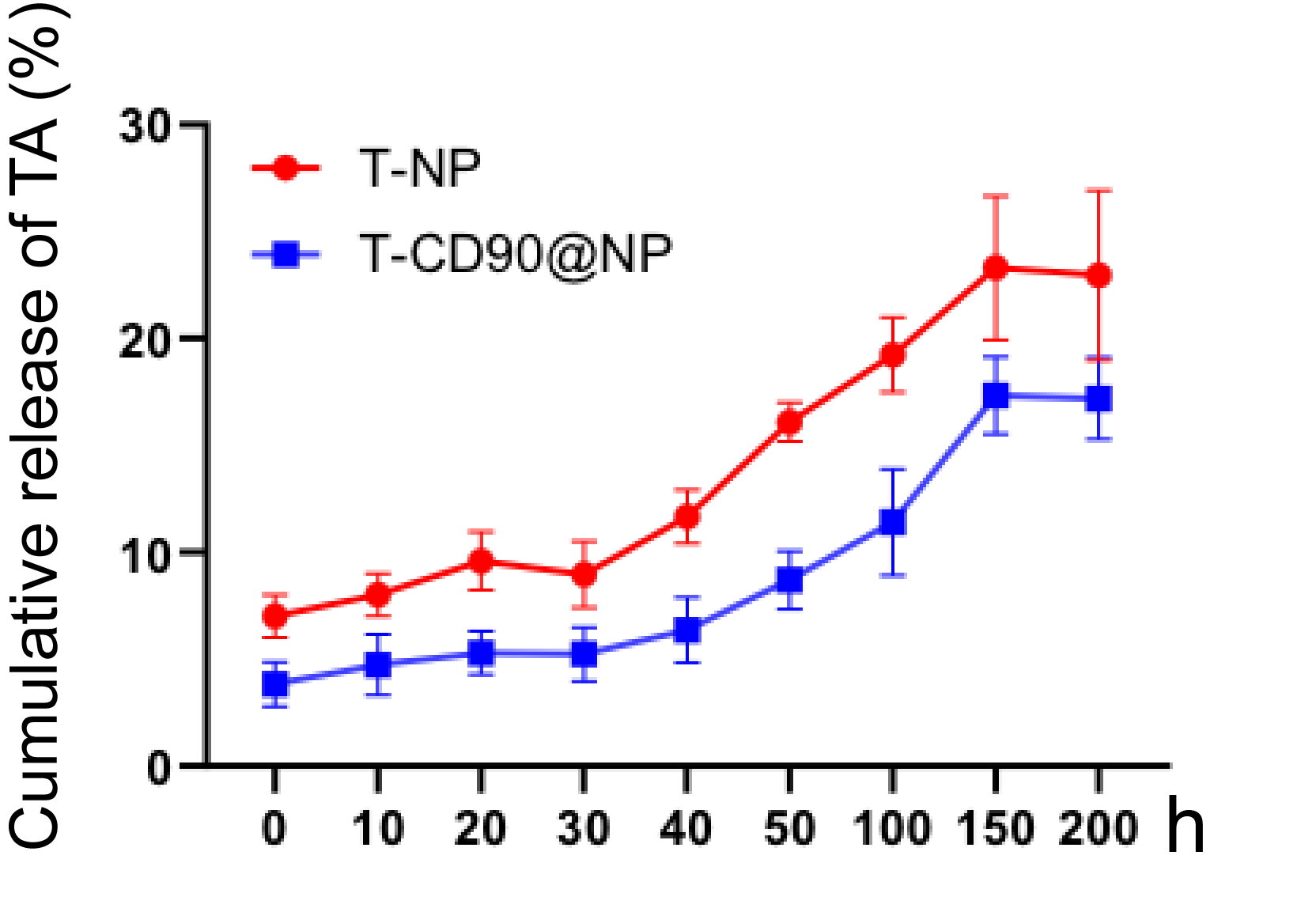

Supplement: Supplementary file 1 — Additional file 1: Figure S1. KEGG analysis showed the enrichment of the FOXO signaling pathway in the OA and T-CD90@NP group comparison. Red represents increased expression, and green represents decreased expression. [file 12951_2022_1367_MOESM1_ESM.png]
